# Home Hygiene and Prevention of Disease

**Published:** 1912-04

**Authors:** 


HOME HYGIENE AND PREVENTION OF DISEASE.—By Norman E. Ditman, M. D., New York. Duffield & Co., 1912. Price not stated. Cloth, 320 pages.

"A medical handbook, containing all the information required for ordinary purposes." That states it tersely and to the point. This book is somewhat unique, both as to the subject matter and the arrangement of its contents, handy for reference. It is arranged alphabetically and embraces a great variety of subjects—almost every condition that may arise in a family or happen to an individual—and more. It deals with flies—well, flies may "happen;" and with "alcoholic drinks"—they "happen" to a fellow all too frequently and have a most deleterious effect, a ruinous effect, in fact, not only on a fellow, but on the race. The avoidance of this one factor of destruction may well come under the head of hygiene or prophylaxis. I wish I could extract and print here the entire chapter on alcohol. It is so eminently sensible and written in such unequivocal terms. Will the people never be enlightened on this subject, and abolish the dreadful "saloon," the one place where more devilment, more killing occurs than any other place on the earth? "All hope abandon, ye who enter here," says Dante, of that other hell. I wish every family in America had this book. It is useful; it is sensible; it is instructive, helpful; it is an eye-opener. Large, clear type, easy reading. I can not do better, I think, than to reproduce a review of this book from the *New York Medical Journal,* March 9, 1912:

"The author of this work very justly remarks in his introduction that first aid to the sick ought to prove as useful to the public as first aid to the injured; and he has used considerable discretion in the kind of information given, so that the reader may render a beginning invalid much valuable assistance without being in a position to do him much harm. In many cases the advice to seek the aid of a doctor is extremely pat, for a great deal of unnecesary suffering is undergone by people neglecting to call in the physician, merely for want of the required suggestion. The book has been boiled down by the omission of much of the padding common in books of the kind; the descriptions are confined to symptoms and to treatment, exactly what is wanted by the worried consultant. The ailments are arranged in alphabetical order, rendering reference quick and easy. There is information of a kind likely to be unsuspected by the casual reader, for example, on cleaning, where useful methods are given for cleaning rooms, woodwork, etc., as well as the skin and body; also on climates, there is quite a little treatise on the various kinds of resorts to be found in the United States. In an article on insanity, which might seem out of place in such a book, there is much good advice given toward preserving the mental health, avoidance of day dreaming, and refusing to give way to passion. Directions are given how to stock a very serviceable home medicine chest. Altogether this is not only a safe, but a valuable book for the practitioner to recommend to his patients, particularly those in sparsely settled districts, where it may save an occasional life and can in no way diminish the physician's prestige."

